# Online Tool for the Assessment of the Burden of COVID-19 in Patients: Development Study

**DOI:** 10.2196/22603

**Published:** 2021-03-31

**Authors:** Esther M J van Noort, Danny Claessens, Catharina C Moor, Carlijn A L Van Den Berg, Marise J Kasteleyn, Johannes C C M in 't Veen, Onno C P Van Schayck, Niels H Chavannes

**Affiliations:** 1 Department of Public Health and Primary Care Leiden University Medical Center Leiden Netherlands; 2 Department of Family Medicine Care and Public Health Research Institute School for Public Health and Primary Care Maastricht University Medical Centre Maastricht Netherlands; 3 Department of Respiratory Medicine Erasmus Medical Center Rotterdam Netherlands; 4 Erasmus Medical Center Rotterdam Netherlands; 5 Department of Pulmonology Franciscus Gasthuis and Vlietland Hospital Rotterdam Netherlands

**Keywords:** COVID-19, patient-reported outcomes, ABCoV tool, monitoring, patient outcome, long-term impact, tool, assessment, online patient platform

## Abstract

**Background:**

The impact of COVID-19 has been felt worldwide, yet we are still unsure about its full impact. One of the gaps in our current knowledge relates to the long-term mental and physical impact of the infection on affected individuals. The COVID-19 pandemic hit the Netherlands at the end of February 2020, resulting in over 900,000 people testing positive for the virus, over 24,000 hospitalizations, and over 13,000 deaths by the end of January 2021. Although many patients recover from the acute phase of the disease, experience with other virus outbreaks has raised concerns regarding possible late sequelae of the infection.

**Objective:**

This study aims to develop an online tool to assess the long-term burden of COVID-19 in patients.

**Methods:**

In this paper, we describe the process of development, assessment, programming, implementation, and use of this new tool: the assessment of burden of COVID-19 (ABCoV) tool. This new tool is based on the well-validated assessment of burden of chronic obstructive pulmonary disease tool.

**Results:**

As of January 2021, the new ABCoV tool has been used in an online patient platform by more than 2100 self-registered patients and another 400 patients in a hospital setting, resulting in over 2500 patients. These patients have submitted the ABCoV questionnaire 3926 times. Among the self-registered patients who agreed to have their data analyzed (n=1898), the number of females was high (n=1153, 60.7%), many were medically diagnosed with COVID-19 (n=892, 47.0%), and many were relatively young with only 7.4% (n=141) being older than 60 years. Of all patients that actually used the tool (n=1517), almost one-quarter (n=356, 23.5%) used the tool twice, and only a small group (n=76, 5.0%) used the tool 6 times.

**Conclusions:**

This new ABCoV tool has been broadly and repeatedly used, and may provide insight into the perceived burden of disease, provide direction for personalized aftercare for people post COVID-19, and help us to be prepared for possible future recurrences.

## Introduction

The COVID-19 pandemic caused by the new coronavirus SARS-CoV-2 swept through the Netherlands from the end of February 2020 and caused over 24,000 hospitalizations and over 13,000 deaths by the end of January 2021 [1]. Although many recovered from the acute infection, damage to the lungparenchyma (portion of the lungs involved in gas exchange) was observed in computed tomography scans of patients who were hospitalized [2], with a subsequent risk of long-term lung damage. Experiences with other coronaviruses also raised a serious concern for long-term *sequelae*. As an example, the Q fever epidemic, which had 4026 cases (in the period 2007-2010) [3], had an extensive aftermath: 20% of patients with acute symptoms subsequently experienced fatigue long after the initial infection was resolved [3]. Adequate follow-up of patients with COVID-19 may reduce the long-term consequences by means of early detection and symptom management.

Monitoring patients who have had a COVID-19 infection is therefore pivotal. In early March 2020, the Lung Foundation Netherlands (a Dutch patient advocacy group) became aware of the need for a better understanding of COVID-19 in the public [4,5]. During the peak of the pandemic (first wave), there was only a limited number of tests for COVID-19 in the Netherlands. Hence, there was a rapid growing requirement for information in those that experienced COVID-19 symptoms but were never tested or medically diagnosed.

The Lung Foundation Netherlands offers a help desk and an online forum for the public for all lung-related questions [6]. In the beginning of the pandemic, Lung Foundation Netherlands was confronted with all kinds of concerns and questions related to COVID-19. To handle these concerns, Lung Foundation Netherlands decided on a structured approach. Since Lung Foundation Netherlands is involved in scientific research [7], it was a logical decision to exploit patient-reported outcome measurements (PROMs) for a better understanding of the symptoms and long-term impacts of COVID-19. However, no tools to report on symptoms and long-term effects of COVID-19 were available yet. On the other hand, PROMs to assess the patient-experienced burden of disease do exist for other chronic conditions. Therefore, we decided upon the development of a COVID-19–oriented tool.

## Methods

### Team

A team of leading medical, technical, and process experts was formed, consisting of the chief executive officer (CEO) of the Lung Foundation Netherlands, the CEO of the Dutch Lung Alliance, experts from the Care and Public Health Research Institute (CAPHRI), pulmonologists from a training hospital (Franciscus Gasthuis and Vlietland) and an academic hospital (Erasmus MC), and the CEO of an eHealth company (Curavista). Because patients infected by COVID-19 are primarily at risk of developing lung damage, the selection of potential tools was narrowed down to tools assessing pulmonary symptoms. It was also considered best to adapt an existing, validated, and well-known tool to be launched as quickly as possible.

### The Assessment of Burden of Chronic Obstructive Pulmonary Disease Tool

The assessment of burden of chronic obstructive pulmonary disease (ABC) tool, used in the monitoring and care for people with chronic obstructive pulmonary disease (COPD) [8-10], was selected as the best-suited tool. The ABC tool was preferred over other tools such as the COPD assessment tool or the COPD control questionnaire for four reasons. First, in the management of chronic conditions such as COPD, there is a paradigm shift from doctor-driven care to patient-centered integrated care with active involvement of and self-management by the patient. The original ABC tool was designed to be used in this transition of facilitating self-management support and shared decision making. As such, it considers symptoms and lifestyle. Second, the ABC tool offers a strong graphical visualization (the status per symptom being scored in *colored balloons*). The balloons are easy to understand, enhancing the long-term patient participation necessary to collect individual long-term data. Third, the ABC tool had previously been adapted for other conditions such as asthma, diabetes, and heart failure [11]. Finally, the tool is already widely used in the Netherlands by both general practitioners (GPs) and hospital specialists in monitoring patients with COPD, and the tool is integrated in the guidelines for regular GP care of patients with COPD [12].

**Figure 1:**
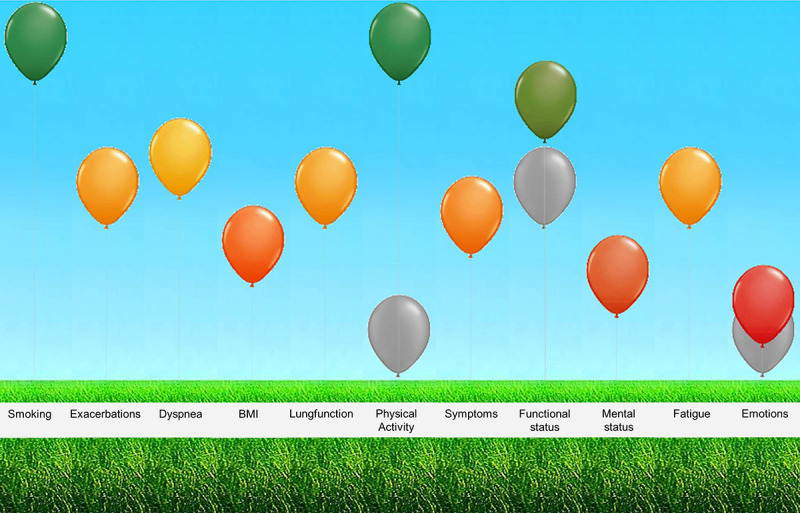
Visualization of the assessment of burden of chronic obstructive pulmonary disease tool.

The ABC tool, developed in 2014, measures the integrated health status of an individual patient with COPD [9]. The self-administered questionnaire consists of 14 statements that evaluate the burden of COPD experienced by patients in five domains (symptoms, functional state, mental state, emotions, and fatigue) and with some objective parameters. An algorithmic computer program visualizes outcomes and provides treatment advice and an index score for future health care costs [10]. Each domain is visualized using balloons: a high green balloon indicates a good score on a particular item, while a low red balloon indicates difficulties on that item (Figure 1).

The previous score appears as a gray balloon and indicates improvement or deterioration. The ABC tool has been validated and proven to be effective and reliable for people with COPD, is proven to be effective in improving quality of life, is perceived as easy to use by both health care providers and patients [8], and is adaptable for other chronic conditions [8-13].

To adjust the ABC tool for use in post–COVID-19 cases, a focused literature search was performed in early April 2020 by the CAPHRI Institute of Maastricht University. This search covered symptoms, complaints, and burden of disease in cases of COVID-19 reported earlier and in reports of the previous coronavirus outbreaks of severe acute respiratory syndrome (SARS) and Middle East respiratory syndrome (MERS).

The most prominent long-term effects of SARS and MERS are chronic fatigue and lung fibrosis, resulting in shortness of breath, dry cough, and decreased physical and mental health [14-19]. Patients with COVID-19 show similar symptoms as well as fever, headache, and chest pain [20-23]. The ABC tool includes all of those symptoms. The aforementioned nonpulmonary symptoms were added to the new tool.

Some patients (29%) with COVID-19 are admitted to the intensive care unit due to acute respiratory distress syndrome (ARDS) [22]. Quite often these patients show signs of a restrictive lung disease (25%) and, in fewer cases (4.5%), obstructive lung disease [21]. The ABC tool includes the most important symptoms of obstructive and restrictive lung disease (Table 1). Furthermore, ARDS can lead to decreased physical and mental health, decreased attention and concentration, and muscle weakness [24]. This warranted the inclusion of symptoms related to postintensive care syndrome and ARDS [24,25]. Physical and mental health are addressed in the ABC tool, but decreased attention and concentration and muscle weakness are not. Since only a subgroup of patients developed ARDS, the expert group considered these three additional symptoms out of scope, and these items were not integrated in the assessment of burden of COVID-19 (ABCoV) tool.

**Table 1:** Modification of symptoms from the ABC tool to the ABCoV tool.

Symptoms	ABC^a^ tool	ABCoV^b^ tool
Smoking	Included	Included
Exacerbation	Included	Excluded
Shortness of breath	Included	Included
BMI	Included	Included
FEV_1_^c^	Included	Included
Physical exercise	Included	Included
Physical well-being: walking, stairs, dishwashing	Included	Included
Symptoms like cough or phlegm production	Included	Included
Mental health due to lung problems	Included	Included
Fatigue	Included	Included
Social well-being, engagement in social activities	Included	Included
Chest pain	—^d^	Added
Dizziness	—	Added
Headache	—	Added
PTSD^e^ screening	—	Added
Open text	—	Added

^a^ABC: assessment of burden of chronic obstructive pulmonary disease.

^b^ABCoV: assessment of burden of COVID-19.

^c^FEV_1_: forced expiratory volume in the first second of expiration.

^d^Item was not in the original ABC tool.

^e^PTSD: posttraumatic stress disorder.

To gain an understanding of the impact of COVID-19 on mental health [24], an additional screening question was added to determine whether patients had traumatic experiences related to COVID-19 infection. If indicated so, the first five questions from the Global Psychotrauma Screen (GPS) [26] are presented to evaluate the risk of developing posttraumatic stress disorder. If not, all questions from the GPS are left out.

Because of the limited information on the long-term sequelae of COVID-19 at that time, it was decided to be overly inclusive. Therefore, only one item (exacerbation) from the original ABC tool for COPD was excluded, as it was not described at all in the literature on COVID-19 at the time of development. The lifestyle items were included as well because lifestyle seemed to be an important risk factor for hospitalization [27] and in influencing outcomes [28]. We included all items from the literature search and additionally offered an *open text field* for all other possible symptoms. This offers the best possibility to get better insight in the incidence of the different symptoms in a new disease like COVID-19. A full overview of the original ABC tool and new ABCoV tool is presented in Table 1. Multimedia Appendix 1 shows the questionnaire, known as the ABCoV tool, that is currently in use.

### Algorithm

**Figure 2:**
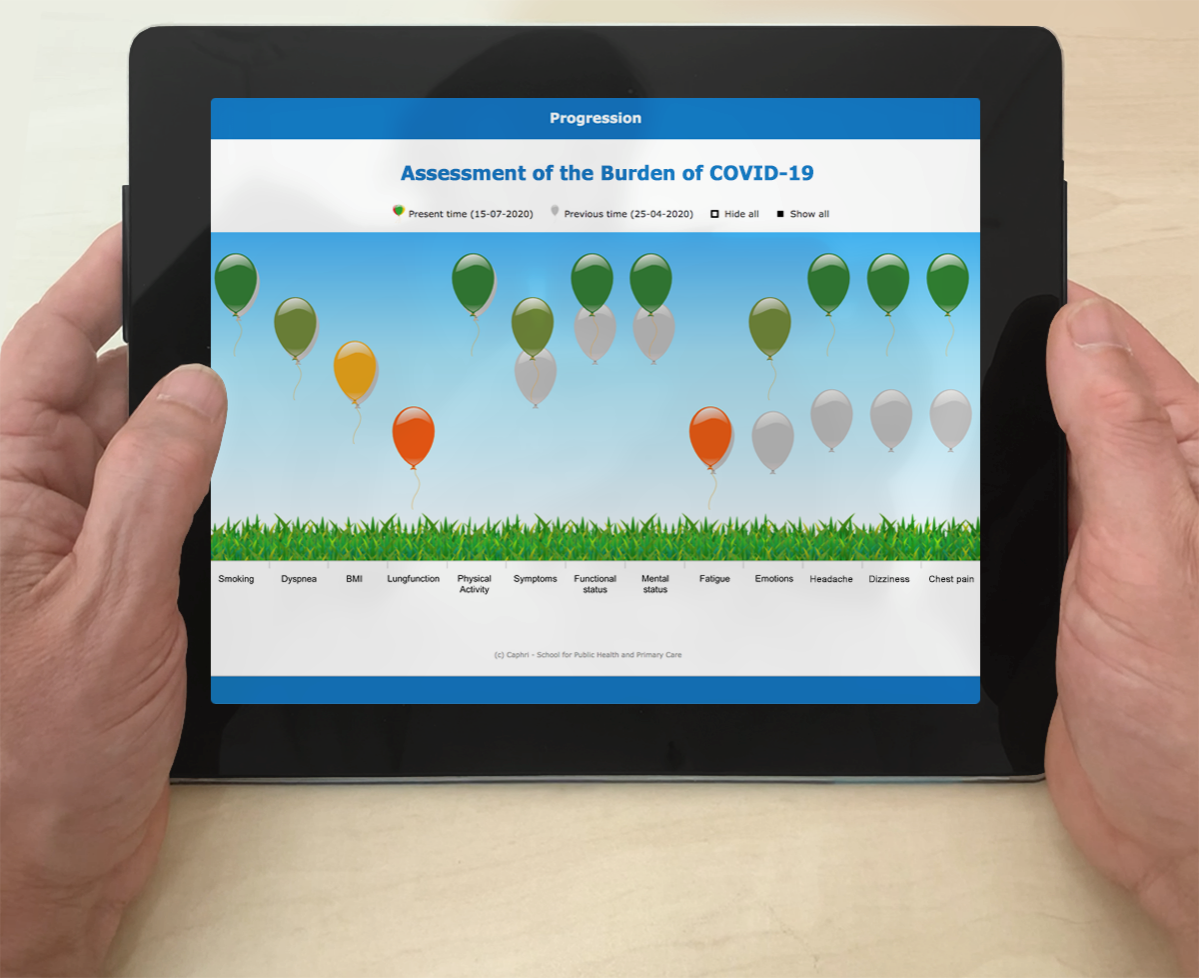
Visualization of the ABCoV tool. ABCoV: assessment of burden of COVID-19; FEV1: forced expiratory volume in the first second of expiration.

Patient responses to the ABCoV tool are translated into balloons with a certain height and color (Figure 2). The height and color of each balloon is determined by the answers to questions. Answers that point toward *no burden of disease* generate high-floating green balloons, and answers that point toward *heavy burden of disease* generate a red balloon down to the ground. Orange, yellow, and light green balloons represent intermediate answers to *burden of disease*. The original algorithm for COPD was unaltered for every item except BMI; optimal BMI ranges differentiate for people with COPD from that of the general population. For the ABCoV tool, the optimal BMI range of the general population was used (<18.5 underweight, 18.5-25 normal weight, 25-30 overweight, >35 obese). A 7-point Likert scale was used for all items in the ABCoV tool other than risk factors. Newly included questions in the ABCoV tool are scored on a visual analog scale ranging from 0 to 10. The ABC tool generates treatment recommendations and an index score assigning the overall burden to one of three categories: low, medium, or high. More research is needed to implement both of these in the ABCoV tool, so they have been omitted for now. The expert group decided not to impose a specific frequency of use or time window since the use of the ABCoV tool is patient-driven. Patients can fill out the ABCoV tool once a day, and there are no reminders.

### Online eHealth Platform

The expert group chose to roll out the tool using the Curavista.health platform, a certified modular platform (NEN7510, ISO27001, CE class I MDD, Health Insurance Portability and Accountability Act compliant), which has the ABC tool already in place and is used by patients, GP practices, and hospitals. The platform is available in six languages, and other languages are being implemented. As such, the tool is scalable.

The ABCoV tool was incorporated, the algorithm validated and tested, and its content cleared by the CAPHRI institute. The preproduction environment was tested by 3 patients. They suggested to add an explanation that describes the purpose of the ABCoV tool and why this was adopted.

### Enrollment

During the first months of the pandemic, only limited testing was available for COVID-19 in the Netherlands. The Lung Foundation Netherlands received many questions from people who experienced COVID-19 symptoms, with or without having been tested or medically diagnosed [4]. Both may benefit from the ABCoV tool to monitor their progression and to get tested if their symptoms worsen or do not subside. Therefore, it was decided to enable registration for everyone for the tool and self-monitoring via the website *Coronalungsquare* [29]. Registered persons can invite their physician to join them online. A *medical route* is also available by which doctors can invite their patients to join and monitor their post–COVID-19 experience using the ABCoV tool online.

## Results

The ABCoV tool was launched on May 7, 2020. A total of 2162 people (each identified by a unique email address) had downloaded the app and self-registered by January 20, 2021. A total of 1898 (87.8%) people gave permission to analyze their data. The majority of participants were female and between 18-60 years of age (Table 2). A subtotal of 1690 (78.1%) people submitted the first screening questions on how and when they were diagnosed with COVID-19. A total of 892 (47.0%) people responded that the diagnosis was medically confirmed either by testing (n=628) or by a medical doctor without testing (n=262). Only 2 people could not recollect how they were diagnosed. A total of 472 people did not answer this question (Table 2). Additionally, 456 patients, diagnosed by pulmonologists at a large teaching hospital (Franciscus Gasthuis and Vlietland) or at one of three academic hospitals (Erasmus Medical Center, Leiden University Medical Center, or Amsterdam University Medical Center), were enrolled for the use of the ABCoV tool, and more GPs and hospitals will do so in the coming months.

Participants can log in with their personal account and submit the ABCoV tool again. Participants do not receive reminders. The majority of the participants submitted the questionnaire once. After submitting the ABCoV 19 tool once, 23.5% (356/1517) of participants submitted the ABCoV tool again (see Table 3).

**Table 2:** Profile of registered users (self-registration).

Demographics		Registered users (N=2162), n (%)
Permission to analyze data		1898 (87.8)
**Gender^a^**		
	Male	364 (19.2)
	Female	1153 (60.7)
	No answer	381 (20.1)
**Age group (years)^a^**		
	<18	5 (0.3)
	18-40	358 (18.9)
	41-60	507 (26.7)
	61-80	141 (7.4)
	>80	1 (0.0)
	Unknown	886 (46.7)
**Medical diagnosis?^a^**		
	Yes	892 (47.0)
	No	534 (28.1)
	No answer	472 (24.9)
**If yes, how were you diagnosed?^b^**		
	By test	628 (70.4)
	By doctor	262 (294)
	Unknown	2 (0.2)

^a^Percentages are based off those who consented (n=1898).

^b^Percentages are based off those who were medically diagnosed (n=892).

**Table 3:** Number of individual submissions in the assessment of burden of COVID-19 tool.

Number of times submitted	Individuals (n=1517), n (%)
One time	1517 (100)
Two times	356 (23.5)
Three times	206 (13.6)
Four times	134 (8.8)
Five times	98 (6.5)
Six times	76 (5.0)
Seven times	57 (3.8)
Other	441 (29.1)

## Discussion

The ABCoV tool was created by an expert team to monitor patients with a (suspected) COVID-19 infection. This tool may detect problems with patients’ physical and psychological health, their social life, and their lifestyle risk factors at an early stage. The tool can be used to study the long-term patient-experienced burden of COVID-19 and provide the insights needed to drive optimal treatment. Obviously, this approach has its limitations. People may not always be aware of a COVID-19 symptom or differentiate it from other causes. This bias can potentially be reduced substantially by asking participants how and when they were diagnosed with COVID-19, by including a large number of participants, and by asking them to submit the ABCoV tool multiple times. We tried to address all possible items; all items from the original ABC tool, except one (exacerbations), were included. We added all other items mentioned in the literature plus an *open text field* for unforeseen symptoms. The next step is to evaluate the ABCoV tool’s validity, practical use, and user experience. Frequent feedback from patients and health care providers is needed to ensure its optimization.

Finally, the purpose of the ABCoV tool is to support patients with COVID-19. In its present presentation, it is a preliminary tool to be validated and evaluated in the future. Better understanding of COVID-19 symptoms obtained by these longitudinal patient-reported outcomes may enable more insights into the long-term impact and disease burden after an infection with COVID-19 and provide tailored health care in a digital patient-centered environment.

## Appendix

Multimedia Appendix 1 The assessment of burden of COVID-19 tool (not validated in English).

## Abbreviations

ABC: assessment of burden of chronic obstructive pulmonary disease
ABCoV: assessment of burden of COVID-19
ARDS: acute respiratory distress syndrome
CAPHRI: Care and Public Health Research Institute
CEO: chief executive officer
COPD: chronic obstructive pulmonary disease
GP: general practitioner
GPS: Global Psychotrauma Screen
MERS: Middle East respiratory syndrome
PROM: patient-reported outcome measurement
SARS: severe acute respiratory syndrome
