# Supplementary material for: Online Tool for the Assessment of the Burden of COVID-19 in Patients: Development Study
Source: JMIR Form Res. 2021 Mar 31;5(3):e22603. doi: 10.2196/22603 (PMC8015936; doi:10.2196/22603)
Supplement: Multimedia Appendix 1 [file formative_v5i3e22603_app1.pdf]

	Yes	No
**1** Have you experienced the COVID-19 infection as unusually or especially frightening, horrible, or traumatic?	☐	☐<br>Go to 6.
In the past month, have you…		
**2** …had nightmares about the COVID-19 infection or thought about the COVID-19 infection when you did not want to?	☐	☐
**3** …tried hard not to think about the COVID-19 infection or went out of your way to avoid situations that reminded you of the COVID-19 infection?	☐	☐
**4** …been constantly on guard, watchful, or easily startled?	☐	☐
**5** …felt numb or detached from people, activities, or your surroundings?	☐	☐
**6** …felt guilty or unable to stop blaming yourself or others for the COVID-19 infection or any problems the COVID-19 infection caused?	☐	☐

**7** Which category applies to you?	*I have never smoked*<br>Go to 9.	*I quit >1yr*<br>Go to 9.	*I quit 6mos–1yr*<br>Go to 9.	*I quit <6mos*<br>Go to 9.	*I smoke*<br>Go to 8.

8 How many cigarettes or cigars do you smoke daily? ....

9 Test if your BMI is in good range. Weight: .... Length: ....

FEV measures lung capacity. If you do not have this value, you can skip this question. ....

**10** In the last week, how many days have you had moderate physical exercise?	*<1 day*	*1-4 days*	*>4 days*

**11** Do you experience shortness of breath?	*No*	*Yes, with heavy physical exercise*	*Yes, when hurrying on level ground or walking on a slope*	*Yes, people my age faster on level ground and/or I have to stop to catch my breath*	*Yes, I have to stop to catch my breath after a few meters*	*Yes, I am too short of breath to go out of the house, or get dressed*

	Never	*Hardly ever*	*A few times*	*Several times*	*Many times*	*A great many times*	*Almost all the time*
On average, during the past week, how often did you feel:							
**12** Short of breath at rest?	☐	☐	☐	☐	☐	☐	☐
**13** Short of breath doing physical activities?	☐	☐	☐	☐	☐	☐	☐
**14** Concerned about getting a cold or your breathing getting worse?	☐	☐	☐	☐	☐	☐	☐
**15** Depressed (down) because of your breathing problems?	☐	☐	☐	☐	☐	☐	☐
In general, during the past week, how much of the time							
**16** Did you cough?	☐	☐	☐	☐	☐	☐	☐
**17** Did you produce phlegm?	☐	☐	☐	☐	☐	☐	☐

	Not limited at all	*Very slightly limited*	*Slightly limited*	*Moderately limited*	*Very limited*	*Extremely limited*	*Totally limited/ or unable to do*
On average, during the past week, how limited were you in these activities because of your breathing problems:							
**18** Strenuous physical activities (such as climbing stairs, hurrying, doing sports)?	☐	☐	☐	☐	☐	☐	☐
**19** Moderate physical activities (such as walking, house work, carrying things)?	☐	☐	☐	☐	☐	☐	☐
**20** Daily activities at home (such as dressing, washing yourself)?	☐	☐	☐	☐	☐	☐	☐
**21** Social activities (such as talking, being with children, visiting friends/relatives)?	☐	☐	☐	☐	☐	☐	☐

	Never	*Hardly ever*	*A few times*	*Several times*	*Many times*	*A great many times*	*Almost all the time*
How often in the past week did you suffer from:							
**22** Worry?	☐	☐	☐	☐	☐	☐	☐
**23** Listlessness?	☐	☐	☐	☐	☐	☐	☐
**24** A tense feeling?	☐	☐	☐	☐	☐	☐	☐
**25** Fatigue?	☐	☐	☐	☐	☐	☐	☐

In general, during the past week, did you suffer from (VAS-score: not at all – extremely)	
**26** Headaches?	0-10
**27** Dizziness?	0-10
**28** Chest pain or pressure?	0-10

29 Did you experience any symptoms not mentioned? ....
